# Hydride Transfer Limits Hydrogen Evolution Efficiency With Zn Porphyrin Photocatalysts

**DOI:** 10.1002/asia.70665

**Published:** 2026-03-06

**Authors:** Ouissam El Bakouri, Simon T. Clausing, Lluís Blancafort

**Affiliations:** ^1^ Institut De Química Computacional i Catàlisi (IQCC) and Departament de Química Universitat de Girona Girona Spain; ^2^ Department of Chemistry Johannes Gutenberg University Mainz Germany

**Keywords:** ab initio calculations, photocatalysis, porphyrinoids, reaction mechanisms

## Abstract

We performed a computational study on the photocatalytic hydrogen evolution mechanism using a Zn‐based metalloporphyrin (ZnP), water, and a cheap sacrificial donor. Based on previous experiments, the active species is a Zn chlorin (ZnC), formed by photohydrogenation of ZnP. Our calculations favor an electron‐proton‐electron‐hydride (EPEH) photocatalytic cycle that consists of one‐electron photoreduction of ZnC followed by protonation of a bridge carbon and a second photoreduction, leading to a key ZnCH_P4_^−^ intermediate. One‐electron photoreduction increases the aromaticity of the porphyrin rings, which explains the favorable photoreduction steps. The final step is a hydride transfer from ZnCH^−^ to a proton donor like an ammonium cation or water, resulting in hydrogen generation. Although this process is thermodynamically allowed, it has a high kinetic barrier and leads to loss of aromaticity, which limits catalytic efficiency. Hydrogen generation competes with ZnCH^−^ protonation and photohydrogenation. The poor activity of ZnCH_P4_^−^ as a hydride donor may be related to the loss of aromaticity associated with the hydride donation. The results have implications for electrocatalytic hydrogen production using porphyrins, which share a similar common intermediate. Therefore, our work will be useful to improve the molecular design of porphyrin‐based photo‐ and electrocatalysts for hydrogen generation.

## Graphical abstract

The mechanism of photocatalytic hydrogen evolution catalyzed by a Zn‐based metalloporphyrin, using water and a cheap sacrificial donor, is studied computationally. The efficiency of hydrogen formation is limited by the competition with hydrogenation of the porphyrin, which leads to a reduced macrocycle.

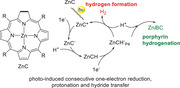

## 1 Introduction

Hydrogen is a promising alternative energy source for fuel cells to generate electricity with minimal emissions [1, 2, 3]. As a building block of base chemicals, it is considered to be a key platform molecule in the future bioeconomy [4]. As we strive toward a “hydrogen‐based economy”, it is essential to develop sustainable hydrogen sources. A common approach to generate it is the use of electrochemical hydrogen evolution reactions, with many of the reported procedures employing metalloporphyrins [5, 6, 7, 8, 9, 10, 11, 12, 13, 14, 15, 16, 17, 18, 19, 20]. Photocatalytic processes for sustainable hydrogen production [21, 22, 23] provide an attractive alternative because they are driven by light instead of electric current. Also here, metalloporphyrins possess unique properties that enable them to mimic the functions of photosynthetic reaction centers and act as photocatalysts [24, 25, 26], such as the ability to absorb light across a wide range of wavelengths within the visible spectrum, tunable electronic and optical properties, and great stability.

**SCHEME 1:**
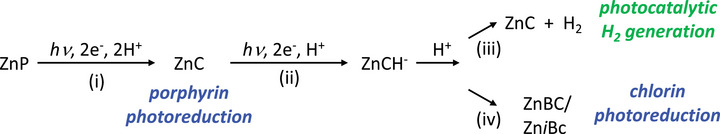
Proposed mechanism for photocatalytic H_2_ production by ZnC.

Usually, photosensitized water reduction requires the use of metal complexes based on e.g. Ni or Co as co‐catalysts. These complexes assist the generation of hydrogen from electrons and protons provided by sacrificial donors and water [27]. However, a potentially more sustainable alternative was discovered in 2017 that does not require any co‐catalyst, redox mediator, or precious metal. It only uses a Zn‐based metalloporphyrin, Zn tetrakis‐(4‐sulfonatophenyl)porphyrin (ZnP), and cheap sacrificial electron donors in aqueous media [28]. Based on experimental data, a mechanism was proposed (Scheme 1) that involves (i) the well‐known photohydrogenation of ZnP to its chlorin analogue (ZnC) [29], which was detected spectroscopically; (ii) the generation of a ZnCH^−^ intermediate after irradiation‐induced two‐electron reduction and protonation of ZnC; and (iii) the formal transfer of a hydride from ZnCH^−^ to a proton from the solution, resulting in H_2_ production and ZnC regeneration. Alternatively, (iv) ZnCH^−^ can also be protonated, resulting in chlorin photohydrogenation to yield a Zn bacteriochlorin (ZnBC) or *iso*bacteriochlorin (Zn*i*BC) that were also detected spectroscopically. Although the turnover number (TON) of H_2_ generation reaches only 1.2, the photocatalytic process appears as an attractive alternative that deserves further study.

In Scheme 1, ZnC has the role of the catalyst, while the electrons come from sacrificial donors like triethylamine and the sulfite anion, and the protons from water. The proposed mechanism poses one intriguing question if one compares the roles of ZnP and ZnC, namely why hydrogen production from ZnC is observed but is suppressed for ZnP. Moreover, the scheme suggests that the low TON can be explained by a competition between photocatalytic H_2_ generation, that is, hydride transfer, and chlorin photohydrogenation, that is, protonation of ZnCH^−^ (steps (iii) and (iv) of Scheme 1). Understanding this competition is key to understanding the low TON.

The photocatalytic process is also interesting in relation to the electrocatalytic generation of hydrogen with free‐base porphyrins [12, 16, 20] or corroles [14] or their analogues containing redox inert metals like Zn^2+^ [20]. In this case the redox transitions involve the porphyrin ligand, and the mechanism can be considered analogous to the photocatalytic process described in Scheme 1. The difference is that in the electrocatalytic H_2_ generation, the electrons are provided by an external bias, whereas in the photocatalytic one they are transferred to the excited porphyrin by a sacrificial donor. Significantly, both processes share the last step, where the fate of the ZnCH^−^ intermediate (or the ZnPH^−^ equivalent, in the electrocatalytic process starting from ZnP) determines the outcome of the reaction.

With these ideas in mind, using quantum chemical calculations, we investigated the mechanism to get a deeper understanding of the process and identify the factors that limit the efficiency of hydrogen production. We have focused on the photocatalytic process involving ZnC and ZnCH^−^ with the aim of identifying the structure of this intermediate and compared the reactivity of ZnC with that of ZnP, which does not show photocatalytic hydrogen formation. Analysis of the changes in aromaticity of the porphyrin ring along the catalytic path provides additional mechanistic insights.

## 2 Results and Discussion

The mechanisms of photocatalytic hydrogen formation and porphyrin/chlorin photohydrogenation have been studied with density functional theory (DFT), using the B3LYP functional (see more Computational Details at the end of the article). Starting from the experimentally studied Zn tetrakis‐(4‐sulfonatophenyl)porphyrin, we have used unsubstituted Zn porphyrin and chlorin as computational models to reduce computational efforts. While this simplification neglects the effect of the electron‐accepting substituents on the reactivity, the use of more general models makes our results more easily extrapolatable to other porphyrins. The results and discussion section is divided into three parts. The first part focuses on the photocatalytic cycle starting from ZnC as the active species, and we explore the mechanism of hydrogen generation. In the second part, we explore the implications of aromaticity for this process. Finally, in the third part, we explore why ZnP does not generate hydrogen photocatalytically and only evolves into ZnC. Even though the conversion of ZnP to ZnC is well known [29], its mechanism has not been clearly established. Thus, we also investigated it computationally.

### 2.1 Hydrogen Production From ZnC

**FIGURE 1:**
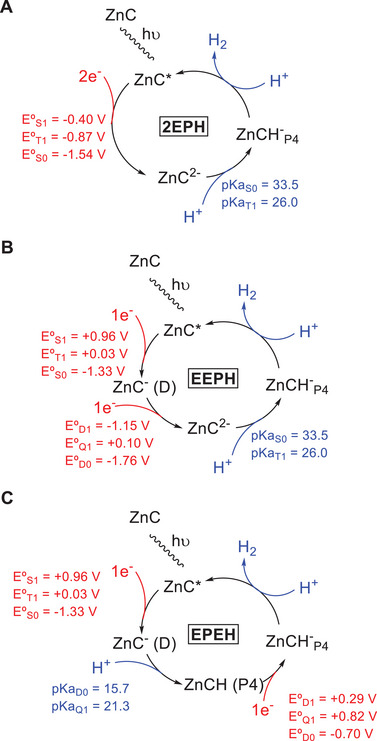
Overview of the three studied mechanisms for photocatalytic hydrogen generation, including the calculated thermodynamic parameters. (**A**) Two‐electron reduction, protonation and hydride transfer mechanism (2EPH) proposed by Knör and coworkers [28], (**B**) consecutive one‐electron reduction, protonation and hydride transfer (EEPH), and (**C**) one‐electron reduction, protonation, one‐electron reduction and hydride transfer (EPEH).

**FIGURE 2:**
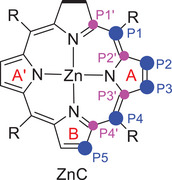
Carbon positions where protonation can take place on ZnC.

Figure 1 displays three possible mechanisms for the photocatalytic cycle, which differ in the way the ZnCH^−^ intermediate is formed. The mechanism proposed by Knör and co‐workers (Figure 1A) consists of excitation of ZnC, reduction of ZnC* with a two‐electron donor (SO_3_^2−^), and protonation of ZnC^2−^ to give ZnCH^−^. In the final step, a hydride from ZnCH^−^ reacts with a proton to give H_2_. We refer to this as a hydride transfer. Therefore, the mechanism is labeled as 2EPH since it involves a sequence of three steps: concurrent transfer of two electrons (2E), proton transfer (P) and hydride transfer (H). There are two alternative mechanisms (Figure 1B,C, respectively). The first one, named EEPH, involves two consecutive one‐electron transfer steps, instead of the concurrent transfer of two electrons in a single step, followed by protonation and hydride transfer. The second one, EPEH, is a sequence made of electron transfer, protonation, electron transfer and hydride transfer. Similar sequential mechanisms have been proposed for the photohydrogenation of ZnP to ZnC [29] and for ligand‐centered electrocatalytic H_2_ generation with porphyrins [12, 14, 16, 20]. Figure 1A provides the calculated reduction potentials for the two‐electron reduction, the initial step of the 2EPH mechanism (see also Table S1). This process has a large negative potential in the ground state of *E*°*_S_*_0_ = −1.54 V *vs* the normal hydrogen electrode (NHE), but it becomes more favorable in the triplet and singlet excited states (*E* ^0^ *_T_*_1_ = −0.87 V, *E*°*_S_*_1_ = −0.40 V), making reduction of ZnC to ZnC^2−^ by SO_3_^2−^ thermodynamically possible (*E* ^0^ = −0.93 V vs the NHE for the SO_4_^2−^/SO_3_^2−^ pair) [30]. Next, protonation of ZnC^2−^ can take place at five different positions (P1–P5, Figure 2), and our calculations suggest that the most favorable one is protonation at P4 (see Table S2), since the protonated species ZnCH^−^_P4_ has the highest pK_a_ among all ZnCH^−^ isomers (33.5). This is consistent with the mechanism proposed in Ref. 28. We note that protonation of ZnC^2−^ in the T_1_ state has a similar preference at P4 and P5, with pK_a_ values of 26.0 and 26.1, respectively (Table S2). In addition to these mechanisms, ZnC^2−^ could also be formed from ZnC^−^ through a disproportionation reaction as suggested previously [29], but this is not favorable thermodynamically (∆*G* = 9.8 kcal/mol).

While the proposed mechanism is plausible if two‐electron sacrificial donors such as SO_3_^2−^ are used, the sequential transfer of two electrons can be considered as an alternative when one‐electron donors such as triethylamine (TEA) are used, which gave the highest TON in Ref. 28. In the EEPH mechanism, the first reduction potential (ZnC ⟶ ZnC^−^) is −1.33 V in the ground state and 0.96 V in the S_1_ state. Therefore, reduction of S_1_ is possible with TEA (reduction potential of 0.45 V) [31]. However, the second reduction potential (ZnC^−^ ⟶ ZnC^2−^) is lower. It is −1.76 V in the ground state, which has doublet multiplicity, and −1.15 V and 0.10 V in the doublet and quartet excited states. This reduction potential is too low to be carried out by TEA, making the EEPH mechanism unlikely. In the alternative EPEH mechanism (Figure 1C), the first electron transfer is followed by protonation. Similar to ZnC^2−^, P4 is the most favorable site according to our calculations, with a pK_a_ of 15.7 (see Table S3). Protonation of ZnC^−^ in the excited (quartet) state has the same preference (pK_a_ = 21.3). The second reduction step (ZnCH_P4_ ⟶ ZnCH_P4_^−^) has a potential of ‐0.70 V in the ground state (D_0_), and 0.29 and 0.82 V in the excited doublet (D_1_) and quartet (Q_1_) states. Considering that our calculations underestimate the reduction potentials by approximately 0.2 V (see Table S13 and the accompanying discussion in the Supporting Information), the calculated value of 0.29 V for D_1_ suggests that reduction of this excited state by TEA is possible. In addition, reduction of the Q_1_ state is also thermodynamically possible. This implies that the second reduction step is also photoinduced.

**FIGURE 3:**
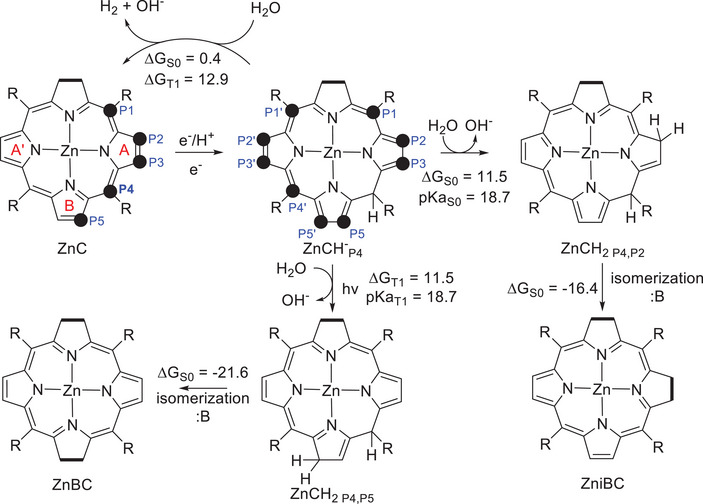
Pathways for hydrogen, ZnBC, and Zn*i*BC formation from ZnC^−^_P4_ in the ground and triplet states. Energies in kcal/mol, B is the base (triethylamine, triethanolamine, sulfite) assisting the isomerization processes.

Following Scheme 1, when ZnCH^−^_P4_ is formed, hydride transfer to a proton donor finally leads to hydrogen formation. However, the efficiency is small and there is experimental evidence that tetrahydroporphyrin species such as ZnBC or Zn*i*BC are formed. These facts reveal that competing reactions take place. We have investigated this in the ground and triplet states (see Figure 3), taking water as the proton donor. According to our computations, formation of hydrogen by hydride transfer to water is approximately thermoneutral in the ground state (Δ*G* = 0.4 kcal/mol), and endergonic in the triplet state (Δ*G* = 12.9 kcal/mol), which implies that this step occurs without need of illumination. Apart from hydrogen formation, a second protonation on ZnCH^−^_P4_ can occur leading to a tetrahydroporphyrin isomer, that is, ZnCH_2_. There are multiple sites prone to protonation (Figure S1), and the computations suggest that the most favorable one in the ground state is position 2, leading to ZnCH_2,P4,P2_ (Δ*G* = 11.5 kcal/mol, which corresponds to pK_a_ = 18.7, see Table S4). ZnCH_2,P4,P2_ can then isomerize to Zn*i*BC in the presence of a base, such as the amines or sulfites that are also present experimentally. This suggests that amines and sulfites also act as bases in addition to electron donors. In turn, protonation of ZnCH^−^_P4_ in the triplet state would take place preferably at P5’ (pK_a_ = 18.7, see Table S4), and the resulting ZnCH_2,P4,P5_ species can isomerize to ZnBC.

**FIGURE 4:**
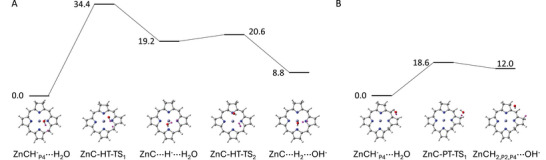
B3LYP/6‐311++G** free energy profiles for (**A**) hydrogen formation and (**B**) ring protonation from the reaction of ZnCH^−^_P4_ with water. The relevant hydrogen atoms are highlighted in purple.

To get further insight into the competition between hydrogen formation and chlorin reduction, the reaction free energy profiles for the two processes have been calculated, including the corresponding transition structures (TS) (see Figure 4). According to the calculations, hydrogen formation (panel A) is a two‐step process where the first step is hydride elimination leading to a trimolecular ZnC···H^−^···H_2_O complex, and the second one is the reaction between the hydride ion and water to give hydrogen and hydroxyl ion. The rate‐determining step is the first one, with a barrier of 34.4 kcal/mol. While this barrier is prohibitively high, one needs to consider that the calculations are approximate. They yield high barriers because only bulk solvation has been considered to increase computational efficiency, neglecting the effect of neighboring water molecules or pH conditions that will facilitate the reaction. In turn, protonation of ZnCH^−^_P4_ is a one‐step process with a barrier of 18.6 kcal/mol (panel B). In spite of the approximate character of these calculations, they show that ZnC^−^_P4_ is a poor hydride donor and that ring protonation is kinetically favored, offering an explanation for the low efficiency of the photocatalytic hydrogen formation cycle observed experimentally. This picture is confirmed by using a stronger proton donor like the ammonium (NH_4_^+^) cation, which is a model for protonated TEA present in the medium of the original experimental work. With NH^+^ as proton donor, H_2_ formation takes place in a single step with a barrier of 29.3 kcal/mol, while ring protonation has a lower barrier of 5.9 kcal/mol (see Figure S2). Similar to the water case, ring protonation is more favored than H_2_ formation. This supports the view that the lack of efficiency of H_2_ formation is primarily determined by the poor hydride donor capacity of ZnCH^−^_P4_, and the acidity of the proton donor plays only a secondary role.

### 2.2 Aromaticity Implications

In this section, we discuss the changes in aromaticity of the macrocycle along the EPEH cycle (Figure 1C). We consider the local aromaticity of the pyrrole rings, in conjunction with the global aromaticity of the system. We rely on a suite of electronic indices, namely the multicenter index (MCI), AV_min_ (an MCI version for larger rings), and electron density of delocalized bonds (EDDB), as our analytical tools to gauge the aromatic character (detailed information is available in the Computational Methods section). Specifically, MCI and EDDB serve to assess the local aromaticity of the pyrrole rings, while AV_min_ and EDDB evaluate the extent of global aromaticity. In the case of EDDB, the density of delocalized bonds is integrated to obtain the number of delocalized π‐electrons participating in specific pathways. This indicator is denoted as *δ*. Analysis of the global aromaticity requires the definition of an annulenic circuit of 18 atoms, for which three different options were considered in ZnC (see Figure S3) [32, 33, 34].

We first consider the effect of reduction on the aromaticity of ZnC. The local *δ* and MCI descriptors show that there is an overall increase of aromaticity of the pyrrole rings (Tables S5 and S6). The average value of *δ* increases from 1.329 for ZnC to 1.843 (ZnC^−^), and that of MCI from 0.026 (ZnC) to 0.030 (ZnC^−^). In contrast to this, the global aromaticity decreases upon reduction, as shown by the AV_min_ values for ZnC and ZnC^−^ along the three possible circuits (Table S8). Turning to the reduction of ZnC^−^ to ZnC^2−^, it is accompanied by a further increase of the average local MCI index from 0.030 to 0.033. Overall, these results suggest that an average increase of the local pyrrole aromaticity facilitates the reduction of ZnC to ZnC^−^ and ZnC^2−^.

Turning to the protonation step, the results of the global EDDB and AV_min_ indices for the protonated species ZnCH_PX_ (Tables S7 and S8) are dependent on the choice of the circuit, which makes them difficult to interpret. For these reasons, we center on the local aromaticity indices (Tables S5 and S6). Here, neither the EDDB nor the MCI local aromaticity indices perfectly reproduce the trend found in the pK_a_ calculations. In particular, the aromaticity of ZnCH_P1_ does not correlate with its stability, since this is the first and second most aromatic species with the EDDB and MCI indices, but P1 is the fourth least favored protonation position according to the calculated pK_a_. This indicates that factors other than aromaticity determine the protonation preference. However, analysis of the local aromaticity still allows for useful insights. First, the EDDB and MCI indices both show that protonation of the P2, P3 and P5 positions drastically reduces the aromaticity of the corresponding pyrrole ring, while protonation at P4 results in only small changes in the pyrrole ring aromaticity (a small reduction in the EDDB case, and a small increase according to the MCI). This is consistent with the preference for protonation of P4 found in the pK_a_ calculations. Finally, the preference for P4 over P1 protonation is correctly indicated by the MCI index.

Turning to the two last steps of the cycle, the average MCI local index increases during the reduction of ZnCH_P4_ to ZnCH^−^_P4_ from 0.033 to 0.037 (Table S6), consistent with what is observed in the previous reduction steps. Significantly, the local aromaticity of ZnCH_P4_^−^ is substantially higher than that of the initial ZnC species (MCI = 0.037 vs. 0.026). This may explain why the last step of the catalytic cycle, which involves a hydride transfer from ZnCH^−^_P4_ to a water molecule, has such a high barrier, since the elimination of the hydride group from the porphyrinoid ring is accompanied by a reduction of the aromaticity. We hypothesize that this is one of the key factors limiting the hydride transfer step, and thus the overall photocatalytic efficiency.

### 2.3 Photohydrogenation of ZnP

Knör and coworkers suggested that the active photocatalytic species is ZnC instead of ZnP. This means that first ZnP must be reduced to ZnC. In fact, this is a widely acknowledged process that occurs under light and in the presence of electron donors [29]. We have carried out a computational investigation to shed light on this mechanism.

**FIGURE 5:**
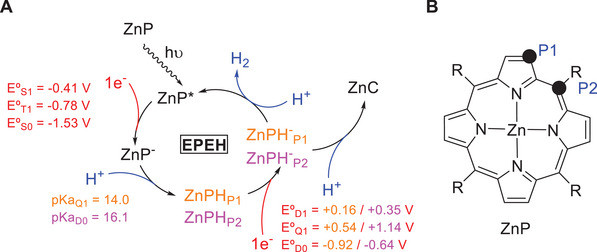
(**A**) EPEH mechanism proposed for the reduction of ZnP to ZnC. (**B**) Carbon positions where protonation can take place on ZnP.

We consider the 2EPH, EEPH, and EPEH mechanisms in analogy to what has been described for ZnC, and first focus on the formation of the ZnPH^−^_PX_ intermediate (X = 1,2). The reduction potentials calculated for ZnP are presented in Table S9 (see Table S13 for a comparison with experimental values). The 2‐electron reduction potentials in the T_1_ and S_1_ states are −0.78 and −0.41 V, respectively. These values are similar than those calculated for ZnC, and reduction of ZnP by SO_3_^2−^ following the 2EPH mechanism is equally possible as for ZnC. For one‐electron donors like TEA, the S_1_ reduction potential of ZnP is 0.95 V, virtually the same as for ZnC, making reduction by TEA possible. Similarly to ZnC, the reduction potential of ZnP^−^ lies between −1.77 and −0.27 V for the ground and excited states, making the EEPH mechanism not plausible. Turning to the EPEH mechanism, after formation of ZnP^−^, the calculated pK_a_ values favor protonation at the methine bridge carbon, which we label as P2, over the pyrrolic P1 position (see Figure 5B for the labeling). The pK_a_ values are 16.1 and 13.5, respectively, see Table S10. Protonation should therefore result in formation of the ZnPH_P2_ intermediate. The calculated reduction potentials of this species to give ZnPH^−^_P2_ are 0.35 and 1.14 V in the doublet and quartet excited states, respectively (see Table S9), confirming that reduction by TEA is thermodynamically possible. Along the EPEH path, the reduction steps are associated with small increases of the aromaticity, with the average local MCI index increasing from 0.025 to 0.027 (ZnP to ZnP^−^) and from 0.031 to 0.032 (ZnPH_P2_ to ZnPH^−^_P2_) (see Table S12).

**FIGURE 6:**
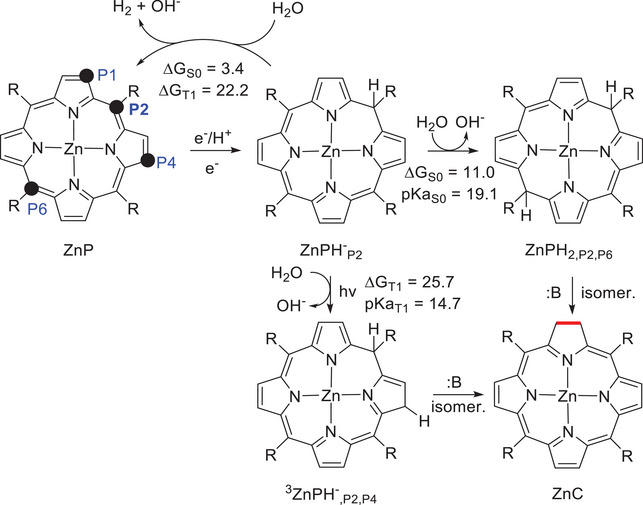
Pathways for hydrogen *vs* ZnC formation from ZnP in the ground and triplet states. Energies in kcal/mol, B stands for the base (triethylamine, triethanolamine, sulfite) assisting the isomerization processes.

Turning to the fate of ZnPH^−^_P2_, pK_a_ calculations indicate that the most favorable position for protonation is the methine bridge position P6 (see Table S11, Figure S4 for labeling). Similar to what we have described for ZnCH^−^_P4_ in Figures 3 and 4 there is competition between hydrogen formation and further protonation of the reduced porphyrin. The formation of hydrogen by reaction with water is thermodynamically favored compared to protonation and ZnPH_2,P2,P6_ formation, with Δ*G_r_* being equal to 3.4 and 11.0 kcal/mol, respectively (see Figure 6). As in ZnCH^−^_P4_, the observed preference for protonation can be explained on the basis of the calculated barriers for the two processes using water as proton donor, which are 35.1 kcal/mol for hydrogen generation (barrier for the rate‐determining hydride elimination), and 17.1 for ring protonation (see Figure S5). With NH_4_^+^ as proton donor, the barriers are lowered to 31.1 and 5.2, respectively (Figure S5). Similar to the ZnC case, calculations of the local MCI indices suggest that the high barriers for hydride elimination are related with the decrease of aromaticity found when going from ZnPH^−^_P2_ to ZnP, of 0.032 to 0.025 (Table S12).

The results for the competition between final hydrogen formation and ring protonation are summarized in Table 1. With both proton donors, H_2_O and NH_4_^+^, ring protonation is kinetically favored (barriers of 18.6 and 17.1 kcal/mol for water, and 5.9 and 5.2 kcal/mol for ammonium proton donor), and the preference over hydrogen formation is more pronounced for ZnPH^−^_P4_ (17.1 vs. 35.1 kcal/mol for water proton donor) than for ZnCH^−^_P4_ (18.6 vs. 34.4 kcal/mol). This is in line with the experimental observation of hydrogen formation for the ZnC catalyst, but not for ZnP.

**TABLE 1:** Summary of rate‐determining free energy barriers (Δ*G^≠^*), in kcal/mol, for the H_2_ formation and ring protonation reactions of the ZnCH^−^_P4_ and ZnPH^−^_P4_ substrates, with H_2_O and NH_4_^+^ as proton donors.

	H_2_O proton donor			NH_4_^+^ proton donor	
Substrate	H_2_ formation	Ring protonation		H_2_ formation	Ring protonation
ZnCH^−^_P4_	34.4		18.6	29.3	5.9
ZnPH^−^_P4_	35.1		17.1	31.1	5.2

## 3 Conclusions

Our calculations for the photocatalytic formation of hydrogen with ZnC as a catalyst suggest that it follows an EPEH cycle consisting of one‐electron photoreduction, protonation, a second photoreduction, and hydride transfer to water, leading to H_2_ formation. The latter step involves a key ZnCH^−^_P4_ intermediate. It goes through the dissociation of the hydride from the chlorin ring, followed by proton abstraction from water. It has a large energy barrier, which makes ZnCH^−^_P4_ a poor hydride donor. Moreover, the hydride transfer competes with protonation of the intermediate, leading to photohydrogenation and formation of an iso‐bacteriochlorin, suggesting that the low TON found experimentally is due to the poor hydride transfer activity of the intermediate. This model also explains why photocatalytic hydrogen formation is not observed with ZnP, since its photohydrogenation is even more favored kinetically over hydrogen formation than in the ZnC case. These conclusions are independent of the strength of the proton donor, since they are found both for water and the ammonium cation as a model for protonated TEA.

The aromaticity of the pyrrole rings plays a role in the different steps of the photocatalytic cycle. Pyrrole aromaticity is increased during the one‐electron photoreduction steps, consistent with the facile photoreduction of the porphyrin and chlorin rings. Pyrrole ring aromaticity is also one of the factors that determines the selectivity of the first protonation step, favoring protonation at the bridge carbon and formation of ZnCH^−^_P4_. Finally, the poor hydride transfer activity of ZnCH^−^_P4_ is consistent with the loss of aromaticity that occurs after elimination of a hydride from the chlorin ring.

Our calculations are also helpful to understand electrocatalytic hydrogen generation using porphyrins. The proposed photocatalytic [28] and electrocatalytic cycles are similar and involve intermediates like ZnCH^−^_P4_ and ZnPH^−^_P4_. It has been argued that molecular design for these processes is not straightforward, and that thermodynamics are not enough to accurately predict which molecular catalytic efficiency [35]. Our calculations on the hydride transfer step show the importance of kinetics, indicating how the mechanistic picture of the catalytic cycles can be completed, and suggesting aromaticity as a further tool of rationalization. Our results, therefore, contribute to improving the conceptual framework for molecular design of porphyrin‐based catalysts.

### 3.1 Computational Methods

**FIGURE 7:**
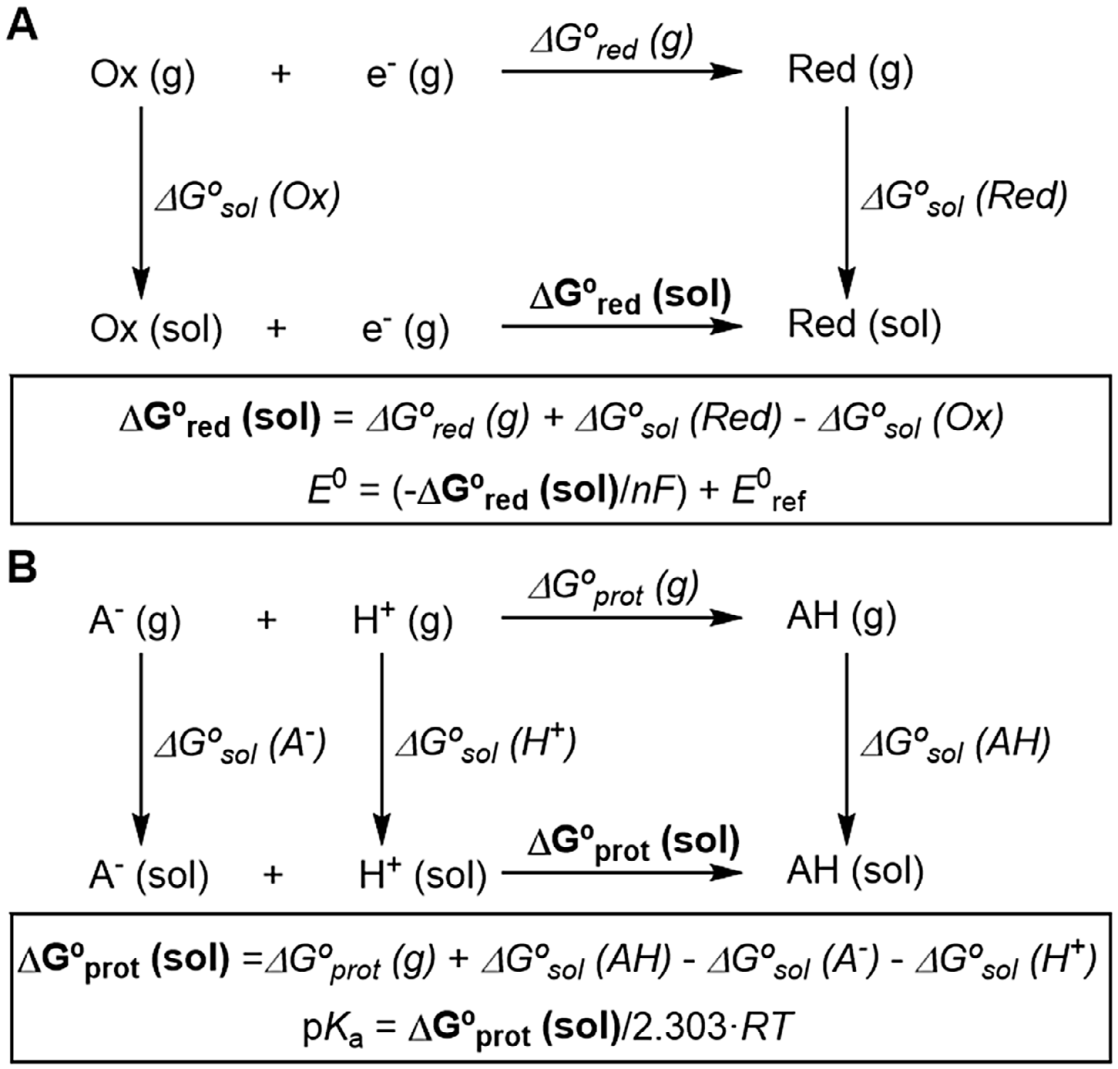
Born–Haber thermodynamic cycle for the calculation of the free energy of electron reduction (**A**) and protonation (**B**).

For the pK_a_ and *E* ^0^ calculations, all molecular geometries were optimized at the B3LYP/6‐31+G(d) level using Gaussian 16 software [36]. The solvent corrections were computed using the SMD continuum solvation model [37] at the B3LYP/6‐311++G(d,p) optimized geometry, where water was used as the solvent. The pKa and reduction potential values were calculated using the Born–Haber thermodynamic cycles (Figure 7). The electrode reference taken for the reduction potentials was the standard hydrogen electrode, with an absolute value of *E* ^0^_ref_ = 4.28 V [38]. This is the recommended value for the calculation of reduction potentials with SMD [39]. This approach was benchmarked by calculating the reduction potentials of TPP and comparing them with experimental reference values. It yields errors of −0.16 and −0.28 V for the one‐ and two‐electron reduction potentials, see details in Table S13. The absolute value of the proton energy in solution was taken as 265.9 kcal/mol, which is the value used for the parametrization of SMD [37, 40, 41]. Triplet (T_1_) states were calculated with triplet multiplicity DFT.

The free energy profiles for the hydrogen formation and ZnP/ZnC hydrogenation steps (Figures 4 and S5) were obtained from minima and transition structure optimizations and the corresponding frequency calculations at the B3LYP/6‐311++G(d,p) level with SMD continuum solvation (water) and D3 empirical dispersion [42]. Note that the thermodynamic values displayed in Figures 4 and S5 for the reaction steps differ from the ones in Figures 3 and 6 because the energies in Figures 4 and S5 are obtained from a single calculation for the whole reacting system, whereas those in Figures 3 and 6 correspond to the sum of the energies calculated separated for every molecule.

Aromaticity was evaluated in terms of electronic indices for which we used the multicenter index (MCI) [43], AV_min_ [44] and the Electron Density of Delocalized Bonds (EDDB) method [45]. MCI quantifies electron delocalization among multiple centers in a molecule. It is based on the molecular wavefunction and uses the electron density overlap integrals between atomic centers. Its expression is:

$$MCI\left(A\right)=\frac{1}{2N}\sum_{P\left(A\right)}\sum_{i_{1},i_{2},\text{…}i_{N}}{n_{i}}_{1}\text{…}{n_{i}}_{N}S_{i_{1}i_{2}}\left(A_{1}\right)S_{i_{2}i_{3}}\left(A_{2}\right)\text{…}S_{i_{N}i_{1}}\left(A_{N}\right)$$

where *S*_ij_(*A*) is the overlap of natural orbitals *i* and *j* in the atom A defined in the framework of the quantum theory of atoms in molecules (QTAIM) and, P(A) stands for a permutation operator which interchanges the atomic labels *A*_1_, *A*_2_… *A_N_* to generate up to the *N*! permutations of the elements in the string A. MCI reflects the extent to which electrons are shared among three or more centers, highlighting delocalization and aromaticity. Here we use the normalized MCI (MCI^1/^ *^n^*), where *n* is the size of the member ring, used to consistently compare aromaticity across rings of different sizes by mitigating the size‐dependent bias of the unnormalized MCI. The AV_min_ index, which is an MCI version for large rings, is the minimal absolute value of four‐center MCI values along the ring. Higher MCI and AV_min_ values indicate stronger electron delocalization and often correlate with increased stability of the molecule. MCI and AV_min_ calculations were carried out with the ESI‐3D program [46] using the QTAIM atomic partition and the integration scheme as implemented in the AIMAll package [47]. EDDB was used to get the number of delocalized electrons within a ring (EDDB_P_ denoted as δ in the present manuscript).

## Conflicts of Interest

The authors declare no conflicts of interest.

## Supporting information



The Supporting Information is available free of charge at 10.1002/asia.70665. The Supporting Information contains reduction potentials, pKas, and aromaticity data related to the investigated species. XYZ coordinates with the absolute electronic energies, and absolute Gibbs free energies can be found as a separate text file.
**Supporting File**: asia70665‐sup‐0001‐SuppMat.pdf.

**Supporting File**: asia70665‐sup‐0002‐SuppMat.txt.

## Data Availability

Structures and energy data were obtained with the commercially available Gaussian16 quantum chemistry program. Aromaticity data were obtained with proprietary, non‐shared software. The formulas used to obtain them are provided in the article. Cartesian coordinates of all structures are provided as a text file in the Supporting Information.
