# Supplementary material for: Hydride Transfer Limits Hydrogen Evolution Efficiency With Zn Porphyrin Photocatalysts
Source: Chem Asian J. 2026 Mar 6;21(5):e70665. doi: 10.1002/asia.70665 (PMC12966634; doi:10.1002/asia.70665)
Supplement: Supplementary file 1 — The Supporting Information is available free of charge at 10.1002/asia.70665. The Supporting Information contains reduction potentials, pKas, and aromaticity data related to the investigated species. XYZ coordinates with the absolute electronic energies, and absolute Gibbs free energies can be found as a separate text file. Supporting File: asia70665‐sup‐0001‐SuppMat.pdf. [file ASIA-21-e70665-s002.pdf]

SUPPORTING INFORMATION

Hydride Transfer Limits Hydrogen Evolution Efficiency with Zn Porphyrin Photocatalysts

Ouissam El Bakouri*[a], Simon T. Clausing[b], Lluís Blancafort[a]

[a] Institut de Química Computacional i Catàlisi (IQCC) and Departament de Química, Universitat de Girona, C/ Maria Aurèlia Capmany 6, 17003 Girona, Catalonia, Spain.

[b] Johannes Gutenberg University, Department of Chemistry, Duesbergweg 10-14, 55128 Mainz, Germany

Table of Contents

Table S1. Calculated reduction potentials for the reduction steps in the proposed photocatalytic cycles involving ZnC (see Figure 1). Data for different electronic states are provided. 4

Table S2. Calculated pK_a values for the protonation of ZnC^{2-} in the singlet and triplet states at different sites (see Figure 2 for site labeling). 4

Table S3. Calculated pK_a values for the protonation of ZnC^- at all possible positions in the doublet and quartet states (see Figure 2 for site labeling). 5

Figure S1. Different positions in which the second protonation of $ZnCH^-_{P4}$ can occur. .5

Table S4. pKas related to the protonation of $ZnCH^-_{P4}$ at all possible positions (see Figure S1 for labeling) in the singlet and triplet states. 5

Figure S2. B3LYP/6-311++G** free energy profiles for (A) hydrogen and (B) tetrahydroporphyrin formation from the reaction of $ZnCH^-_{P4}$ with NH_4^+. The relevant hydrogen atoms are highlighted in purple. 6

Figure S3. Three delocalized circuits of ZnC used for the calculation of global aromaticity indices. 6

Table S5. Number of π-electrons that participate in the π-delocalization in each pyrrole ring for ZnC ($\boldsymbol{\delta ZnC}$), ZnC^- ($\boldsymbol{\delta ZnC -}$) and the protonated species ($\boldsymbol{\delta ZnCHPX}$, X = 1-5). $\boldsymbol{\Delta\delta ZnCHPX - ZnC -}$ is the difference between the number of π-electrons in $ZnCH_{PX}$ and ZnC^-. 7

Table S6. MCI (e^-) of each pyrrole ring for ZnC^- and each protonated species ($\boldsymbol{MCIZnCHPX}$, X = 1-5). $\boldsymbol{\Delta MCIZnCHPX - ZnC -}$ is the difference between the π-electrons in the $ZnCH_{PX}$ species and that of ZnC^-. 8

Table S7. Number of π-electrons that participate in the π-delocalization ($EDDB_P$) in the chlorin and bacteriochlorin circuits (Figure S2) for ZnC^- ($\boldsymbol{\delta ZnC -}$) and each protonated species ($\boldsymbol{\delta ZnCHPX}$, X = 1-5). $\boldsymbol{\Delta\delta ZnCHPX - ZnC -}$ is the difference between the number of π-electrons in $ZnCH_{PX}$ and ZnC^-. 9

Table S8. AV_{min} indices characterizing π-delocalization in the chlorin and bacteriochlorin circuits (Figure S2) for ZnC ($\boldsymbol{AVminZnC}$), ZnC^- ($\boldsymbol{AVminZnC -}$) and the protonated species ($\boldsymbol{AVminZnCHPX}$, X = 1-5). $\boldsymbol{\Delta AVminZnCHPX - ZnC -}$ is the difference between the π-electrons in the $ZnCH_{PX}$ species and that of ZnC^-. 10

Table S9. Calculated reduction potentials for the reduction steps in the proposed photocatalytic cycles involving ZnP. Data for different electronic states are provided. 11

Table S10. Calculated pK_a values for the protonation of ZnP^- at all possible positions in the doublet and quartet states. 11

Figure S4. Multiple positions where the second protonation of $ZnPH^-_{P4}$ can occur. 11

Table S11. Calculated pK_a values for the protonation of $ZnPH^-_{P2}$ at all possible positions (Figure S4) in the singlet and triplet states. 12

Figure S5. B3LYP/6-311++G** free energy profiles for (A) hydrogen and (B) dihydroporphyrin formation from the reaction of $ZnPH^{-}_{P2}$ with water. The relevant hydrogen atoms are highlighted in purple. 12

Figure S6. B3LYP/6-311++G** free energy profiles for (A) hydrogen and (B) dihydroporphyrin formation from the reaction of $ZnPH^{-}_{P2}$ with NH_4^+. The relevant hydrogen atoms are highlighted in purple. 13

Table S12. MCI (e^-) of each pyrrole ring for ZnP, ZnP^-, and the protonated species ($\boldsymbol{MCIZnCHPX}$, X = 1-5). $\boldsymbol{MCIZnPHPX-}$ is the difference between the π-electrons in the $ZnCH_{PX}$ species and that of ZnC^-. 14

Comparison of calculated and experimental reduction potentials 15

Table S13. Summary of experimental one-electron reduction potentials for various porphyrins, and computed potentials for TPP for comparison. Values without brackets are referred to the SHE in water, and the values in brackets are the original literature values (see footnotes for details). 16

References 16

Table S1. Calculated reduction potentials for the reduction steps in the proposed photocatalytic cycles involving ZnC (see Figure 1). Data for different electronic states are provided.

	S_0	D_0	T_1	Q_1	S_1	D_1
$ZnC + 2e^- \rightarrow ZnC^{2-}$	-1.54		-0.87		-0.40	
$ZnC + e^- \rightarrow ZnC^-$	-1.33		+0.03		+0.96	
$ZnC^- + e^- \rightarrow ZnC^{2-}$		-1.76		+0.10		-1.15
$ZnCH_{PX} + e^- \rightarrow ZnCH^-_{PX}$						
P1		-1.12		+0.72		-0.11
P1’		-0.85		+0.64		+0.19
P2		-1.09		+0.74		+0.19
P2’		-0.82		+0.88		+0.25
P3		-0.93		+0.80		+0.37
P3’		-1.01		+0.88		+0.22
P4		-0.70		+0.82		+0.29
P4’		-1.13		+0.32		-0.30
P5		-1.21		+0.14		-0.39

Table S2. Calculated pK_a values for the protonation of ZnC^{2-} in the singlet and triplet states at different sites (see Figure 2 for site labeling).

$ZnC^{2-} + H^+ \rightarrow ZnCH^-_{PX}$	S_0	T_1
P1	25.0	18.6
P2	26.1	20.8
P3	28.4	21.9
P4	33.5	26.0
P5	21.6	26.1

Table S3. Calculated pK_a values for the protonation of ZnC^- at all possible positions in the doublet and quartet states (see Figure 2 for site labeling).

$ZnC^- + H^+ \rightarrow ZnCH_{PX}$	D_0	Q_1
P1	14.2	14.6
P1'	*8.2*	*14.5*
P2	14.8	15.2
P2'	*3.1*	*5.8*
P3	14.5	16.6
P3'	*2.7*	*2.1*
P4	15.7	21.3
P4'	*0.6*	*7.5*
P5	12.4	21.0

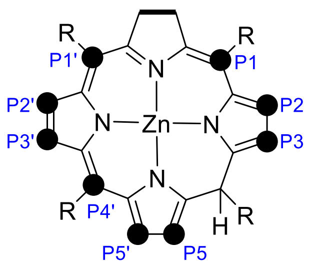

Figure S1. Different positions in which the second protonation of $ZnCH^-_{P4}$ can occur.

Table S4. pKas related to the protonation of $ZnCH^-_{P4}$ at all possible positions (see Figure S1 for labeling) in the singlet and triplet states.

**$ZnCH^-_{P4} + H^+ \rightarrow ZnPH_{2,\ P4,PX}$ (PX)**	S_0	T_1
**P1 (olefin)**	16.3	9.5
**P2 (pyrrole)**	18.7	12.4
**P3 (pyrrole)**	13.9	3.1
**P5 (pyrrole)**	10.1	14.3
**P1' (olefin)**	16.5	5.7
**P2' (pyrrole)**	8.5	15.9
**P3' (pyrrole)**	-3.6	17.7
**P4' (olefin)**	7.1	15.5
**P5' (pyrrole)**	12.5	18.7

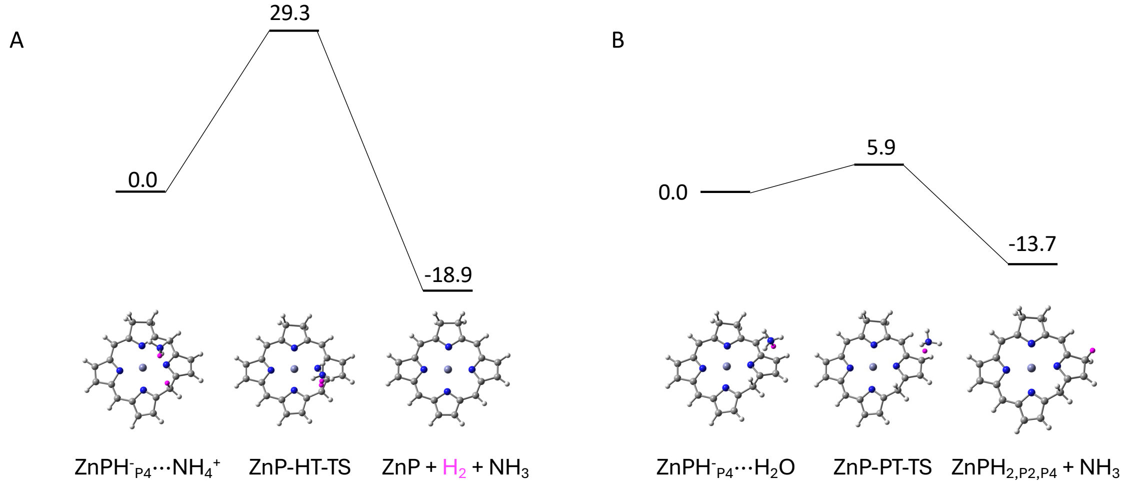

Figure S2. B3LYP/6-311++G** free energy profiles for (A) hydrogen and (B) tetrahydroporphyrin formation from the reaction of $ZnCH^{-}_{P4}$ with NH_4^+. The relevant hydrogen atoms are highlighted in purple.

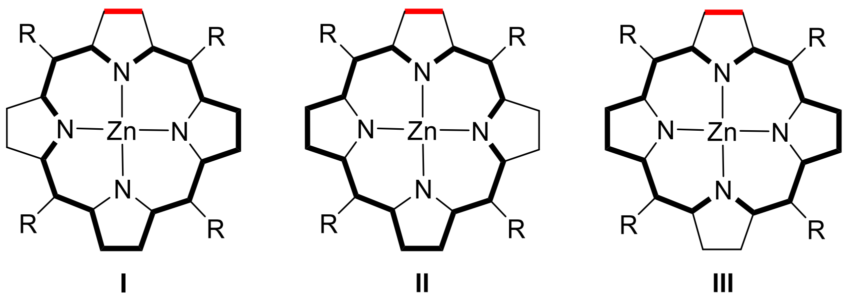

Figure S3. Three delocalized circuits of ZnC used for the calculation of global aromaticity indices.

Table S5. Number of π-electrons that participate in the π-delocalization in each pyrrole ring for ZnC ($\boldsymbol{\delta_{ZnC}}$), ZnC^- ($\boldsymbol{\delta_{ZnC^-}}$) and the protonated species ($\boldsymbol{\delta_{ZnCH_{PX}}}$, X = 1-5). $\boldsymbol{\Delta\delta_{ZnCH_{PX}-ZnC^-}}$ is the difference between the number of π-electrons in $ZnCH_{PX}$ and ZnC^-.

		δ_{ZnC}	δ_{ZnC^-}	$\delta_{ZnCH_{PX}}$	$\Delta\delta_{ZnCH_{PX}-ZnC^-}$
**$ZnCH_{P1}$**	Ring A	1.4248	2.3648	2.4033	0.0385
	Ring A’	1.4243	2.3643	1.9422	-0.4221
	Ring B	1.1391	0.7991	0.7484	-0.0507
	Average	1.3294	1.8427	1.6980	-0.1448
**$ZnCH_{P2}$**	Ring A			0.1841	-2.1807
	Ring A’			1.9112	-0.4531
	Ring B			0.9283	0.1292
	Average			1.0079	-0.8349
**$ZnCH_{P3}$**	Ring A			0.1795	-2.1853
	Ring A’			1.3987	-0.9656
	Ring B			1.274	0.4749
	Average			0.9507	-0.8920
**$ZnCH_{P4}$**	Ring A			2.0395	-0.3253
	Ring A’			1.0216	-1.3427
	Ring B			1.9444	1.1453
	Average			1.6685	-0.1742
**$ZnCH_{P5}$**	Ring A			1.9243	-0.4405
	Ring A’			0.5135	-1.8508
	Ring B			0.1563	-0.6428
	Average			0.8647	-0.9780

Table S6. MCI (e^-) of each pyrrole ring for ZnC^- and each protonated species ($MCI_{ZnCH_{PX}}$, X = 1-5). $\Delta MCI_{ZnCH_{PX}-ZnC^-}$ is the difference between the π-electrons in the $ZnCH_{PX}$ species and that of ZnC^-.

		MCI_{ZnC}	MCI_{ZnC^-}	$MCI_{ZnC^{2-}}$	$MCI_{ZnCH_{PX}}$	$\Delta MCI_{ZnCH_{PX}-ZnC^-}$	$MCI_{ZnCH_{PX}^-}$
**$ZnCH_{P1}$**	Ring A	0.0238	0.0337	0.0428	0.0380	0.0042	0.0441
	Ring A'	0.0238	0.0337	0.0428	0.0302	-0.0035	0.0353
	Ring B	0.0309	0.0237	0.0137	0.0267	0.0030	0.0149
	Average	0.0262	0.0304	0.0331	0.0316	0.0012	0.0314
**$ZnCH_{P2}$**	Ring A				0.0036	-0.0302	0.0032
	Ring A'				0.0285	-0.0053	0.04
	Ring B				0.0239	0.0002	0.0147
	Average				0.0187	-0.0118	0.0193
**$ZnCH_{P3}$**	Ring A				0.0038	-0.0299	0.0031
	Ring A'				0.0247	-0.0091	0.0326
	Ring B				0.0240	0.0003	0.0251
	Average				0.0175	-0.0129	0.0203
**$ZnCH_{P4}$**	Ring A				0.0334	-0.0003	0.0464
	Ring A'				0.0295	-0.0042	0.0227
	Ring B				0.0363	0.0126	0.0417
	Average				0.0331	0.0027	0.0369
**$ZnCH_{P5}$**	Ring A				0.0267	-0.0070	0.0367
	Ring A'				0.0190	-0.0148	0.0169
	Ring B				0.0041	-0.0196	0.0033
	Average				0.0166	-0.0138	

Table S7. Number of π-electrons that participate in the π-delocalization ($EDDB_P$) in the chlorin and bacteriochlorin circuits (Figure S2) for ZnC^- ($\boldsymbol{\delta_{ZnC^-}}$) and each protonated species ($\boldsymbol{\delta_{ZnCH_{PX}}}$, X = 1-5). $\boldsymbol{\Delta\delta_{ZnCH_{PX}-ZnC^-}}$ is the difference between the number of π-electrons in $ZnCH_{PX}$ and ZnC^-.

	**Circuit**	$\boldsymbol{\delta_{ZnC^-}}$	$\boldsymbol{\delta_{ZnCH_{PX}}}$	$\boldsymbol{\Delta\delta_{ZnCH_{PX}-ZnC^-}}$
**$ZnCH_{P1}$**	I	1.9736	0.0635	-1.9101
	II	1.9736	0.0608	-1.9128
	III	**5.0701**	**0.0549**	**-5.0152**
**$ZnCH_{P2}$**	I		0.0623	-1.9113
	II		2.5394	0.5658
	III		**0.0512**	**-5.0189**
**$ZnCH_{P3}$**	I		0.0442	-1.9294
	II		1.9280	-0.0456
	III		**0.0479**	**-5.0222**
**$ZnCH_{P4}$**	I		0.0343	-1.9393
	II		0.0566	-1.9170
	III		**0.0397**	**-5.0304**
**$ZnCH_{P5}$**	I		0.0485	-1.9251
	II		0.0818	-1.8918
	III		**1.6043**	**-3.4658**

Table S8. AV_{min} indices characterizing π-delocalization in the chlorin and bacteriochlorin circuits (Figure S2) for ZnC ($\boldsymbol{AV}_{min_{ZnC}}$), ZnC^- ($\boldsymbol{AV}_{min_{ZnC^-}}$) and the protonated species ($\boldsymbol{AV}_{min_{ZnCH_{PX}}}$, X = 1-5). $\boldsymbol{\Delta AV}_{min_{ZnCH_{PX}-ZnC^-}}$ is the difference between the π-electrons in the $ZnCH_{PX}$ species and that of ZnC^-.

	**Circuit**	$AV_{min_{ZnC}}$	$AV_{min_{ZnC^-}}$	$AV_{min_{ZnCH_{PX}}}$	$\Delta AV_{min_{ZnCH_{PX}-ZnC^-}}$
**$ZnCH_{P1}$**	I	0.401	0.0547	0.0027	-0.0519
	II	0.401	0.0547	0.0033	-0.0514
	III	**0.795**	**0.2066**	**0.0027**	**-0.2039**
**$ZnCH_{P2}$**	I			0.0658	0.0112
	II			0.2658	0.2111
	III			**0.0658**	**-0.1408**
**$ZnCH_{P3}$**	I			0.0472	-0.0075
	II			0.0922	0.0375
	III			**0.0472**	**-0.1594**
**$ZnCH_{P4}$**	I			0.0710	0.0163
	II			0.0193	-0.0353
	III			**0.0368**	**-0.1698**
**$ZnCH_{P5}$**	I			0.0041	-0.0506
	II			0.0041	-0.0506
	III			**0.2609**	**0.0543**

Table S9. Calculated reduction potentials for the reduction steps in the proposed photocatalytic cycles involving ZnP. Data for different electronic states are provided.

	S_0	D_0	T_1	Q_1	S_1	D_1
ZnP + 2e$^-$ → ZnP^{2-}	-1.53		-0.78		-0.41	
ZnP + e$^-$ → ZnP$^-$	-1.29		+0.21		+0.95	
ZnP$^-$ + e$^-$ → ZnP^{2-}		-1.77		-0.27		-1.65
ZnPH$_{PX}$ + e$^-$ → ZnPH$^-_{PX}$						
P1		-0.93		+0.54		+0.16
P2		-0.64		+1.14		+0.35

Table S10. Calculated pK_a values for the protonation of ZnP$^-$ at all possible positions in the doublet and quartet states.

ZnP$^-$ + H$^+$ → ZnPH$_{PX}$	D_0	Q_1
P1	13.5	14.0
P2	16.1	11.4

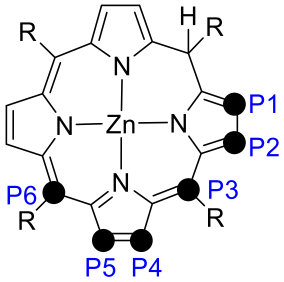

ZnCH$^-_{P4}$

Figure S4. Multiple positions where the second protonation of ZnPH$^-_{P4}$ can occur.

Table S11. Calculated pK_a values for the protonation of $ZnPH^-_{P2}$ at all possible positions (Figure S4) in the singlet and triplet states.

$ZnPH^-_{P2} + H^+ \rightarrow ZnPH_{2, P2,PX}$ (PX)	S_0	T_1
**P1 (pyrrole)**	10.0	10.6
**P2 (pyrrole)**	14.4	13.0
**P3 (olefin)**	13.1	11.0
**P4 (pyrrole)**	-3.2	14.7
**P5 (pyrrole)**	7.6	11.6
**P6 (olefin)**	19.1	2.5

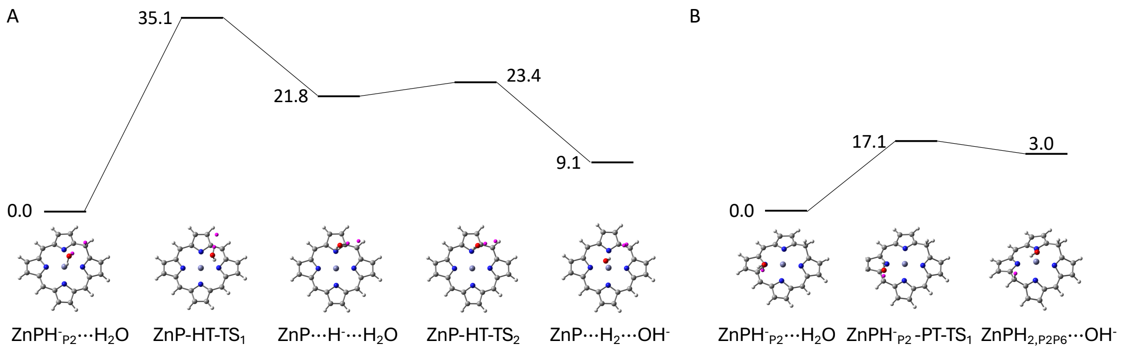

Figure S5. B3LYP/6-311++G** free energy profiles for (A) hydrogen and (B) dihydroporphyrin formation from the reaction of $ZnPH^-_{P2}$ with water. The relevant hydrogen atoms are highlighted in purple.

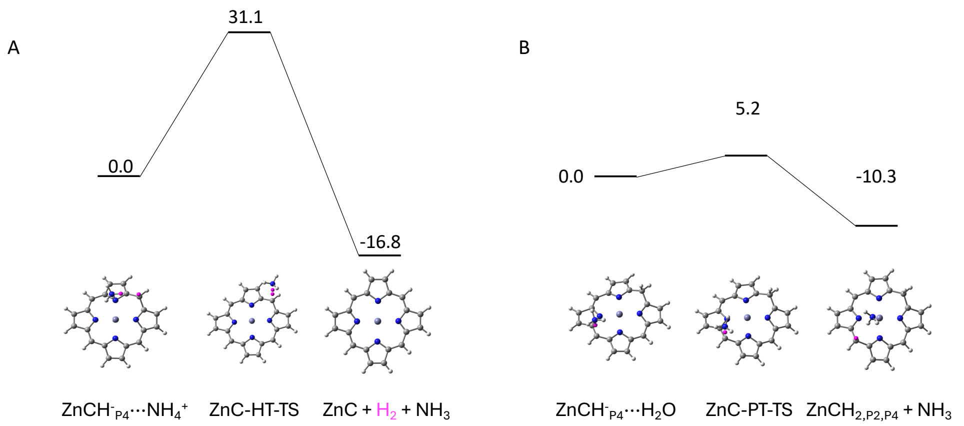

Figure S6. B3LYP/6-311++G** free energy profiles for (A) hydrogen and (B) dihydroporphyrin formation from the reaction of $ZnPH^{-}_{P2}$ with NH_4^{+}. The relevant hydrogen atoms are highlighted in purple.

Table S12. MCI (e^-) of each pyrrole ring for ZnP, ZnP^-, and the protonated species ($\boldsymbol{MCI_{ZnCH_{PX}}}$, X = 1-5). $\boldsymbol{MCI_{ZnPH^-_{PX}}}$ is the difference between the π-electrons in the $ZnCH_{PX}$ species and that of ZnC^-.

		MCI_{ZnP}	MCI_{ZnP^-}	$MCI_{ZnPH_{PX}}$	$MCI_{ZnPH^-_{PX}}$
**$ZnPH_{P1}$**	Ring A	0.0248	0.0190	0.0038	0.0032
	Ring A’	0.0248	0.0343	0.0215	0.0236
	Ring B	0.0248	0.0190	0.0274	0.0331
	Ring B’	0.0248	0.0343	0.0221	0.0146
	Average	0.0248	0.0267	0.0187	0.0186
**$ZnPH_{P2}$**	Ring A	0.0248	0.0190	0.0339	0.0430
	Ring A’	0.0248	0.0343	0.0339	0.0430
	Ring B	0.0248	0.0190	0.0271	0.0200
	Ring B’	0.0248	0.0343	0.0271	0.0200
	Average	0.0248	0.0267	0.0305	0.0315

Comparison of calculated and experimental reduction potentials

Our calculated one-electron reduction potentials for ZnP are -1.29 V and -1.53 V for the first and second reduction, $E_{red}(ZnP/ZnP^{-})$ and $E_{red}(ZnP^{-}/ZnP^{2-})$. While we have not found the experimental values for ZnP, the results can be compared with some literature values for analogue systems (see Table S13). The one-electron reduction potential of free-base tetraphenylporphyrin (TPP) is -1.55 V, measured against the Ag^{+}/Ag electrode in dimethyl formamide (DMF).[1] Using the proposed correction of 0.58 V,[2] the estimated value against the standard hydrogen electrode (SHE) is 0.97 V. The experimental E_{red} of Zn-tetrakis-(4-sulfonatophenyl)porphyrin (ZnTPPS), the compound used in our experimental study of reference,[2] is -1.16 V against the saturated calomel electrode (SCE),[3] whereas that for Zn-tetrakis(N-methyl-4-pyridyl)porphyrin (ZnTMPyP) is -0.85 V.[3] These values translate to -0.92 and -0.61 V *vs* the SHE. The difference between ZnTPPS and ZnTMPyP presumably lies in the total charges of the substituted porphyrin, which are -2 and +2, respectively. As for the second reduction potential, $E_{red}(P^{-}/P^{2-})$, the experimental values corrected *vs* SHE are -1.41 V for free base TPP[1] and -1.16 V for free-base TPPS.[3] For a direct comparison, the calculated potentials for TPP are -1.13 V for $E_{red}(TPP/TPP^{-})$, and -1.69 V for $E_{red}(ZnP^{-}/ZnP^{2-})$, *ie* the calculations underestimate the reduction potentials by approximately 0.2 - 0.3 V.

Table S13. Summary of experimental one-electron reduction potentials for various porphyrins, and computed potentials for TPP for comparison. Values without brackets are referred to the SHE in water, and the values in brackets are the original literature values (see footnotes for details).

	**Experimental**		**Computed**	
**Compound**	**E_{red} (P/P^-) [V]**	**E_{red} (P^-/P^{2-}) [V]**	**E_{red} (P/P^-) [V]**	**E_{red} (P^-/P^{2-}) [V]**
TPP[a]	-0.97<br>(-1.55)	-1.41<br>(-1.99)	-1.13	-1.69
ZnTPPS[b]	-0.92<br>(-1.16)	-	-	-
ZnTMPyP[b]	-0.61<br>(-0.85)	-	-	-
TPPS[b]	-0.82<br>(-1.06)	-1.16<br>(-1.40)	-	-

[a]Ref. SI1; determined in DMF vs Ag^+/Ag in DMF. [b]Ref. SI3; determined in water vs SCE.

References

1) P. Worthington, P. Hambright, R. F. X. Williams, J. Reid, C. Burnham, A. Shamin, J. Turay, D. M. Bell, R. Kirkland, R. G. Little, N. Datta-Gupta and U. Eisner, J. *Bioinorg. Chem.*, 1980, **12**, 281.
2) S. Salzl, M. Ertl and G. Knör, *Phys. Chem. Chem. Phys.*, 2017, **19**, 8141–8147.
3) K. Kalyanasundaram, M. Neumann-Spallart, *J. Phys. Chem.*, 1982, **86**, 5163–5169.
